# Chronic Stress and Astrocyte Dysfunction in Depression: Molecular Mechanisms and Gene Expression Changes

**DOI:** 10.3390/antiox14121464

**Published:** 2025-12-06

**Authors:** Natalia Bochenska, Julia Tomczak, Malwina Lisek

**Affiliations:** Department of Molecular Neurochemistry, Medical University of Lodz, 92-215 Lodz, Poland

**Keywords:** major depressive disorder, depression, astrocytes, glial cells, chronic stress

## Abstract

Major depressive disorder (MDD) is a complex and heterogeneous psychiatric condition with high global prevalence and significant personal and societal burdens. While traditionally focused on neuronal dysfunction, emerging research highlights a critical role for astrocytes—glial cells essential for maintaining brain homeostasis in the pathogenesis of depression. This review explores how chronic stress, a major risk factor for MDD, disrupts astrocyte function through multiple converging mechanisms. We detail the normal physiological roles of astrocytes in synaptic regulation, neurotransmitter cycling, metabolic support, and neurovascular integrity, and examine how these functions are compromised under chronic stress. Key molecular pathways implicated include glucocorticoid receptor (GR) signaling dysregulation, neuroinflammatory responses, glutamate excitotoxicity, oxidative stress, and epigenetic alterations. Evidence from histological and transcriptomic studies in both human postmortem tissue and rodent models reveals consistent changes in astrocyte-specific genes, such as GFAP, SLC1A2, SLC1A3, BDNF, and AQP4, supporting their involvement in depressive pathology. Finally, we discuss therapeutic strategies targeting astrocyte dysfunction—including EAAT2 upregulation, neuromodulation, anti-inflammatory approaches, GR modulation, and glial-focused epigenetic therapies. Understanding astrocyte pathology in the context of chronic stress not only refines our understanding of MDD but also opens novel avenues for treatment development.

## 1. Introduction

Depression is a widespread and debilitating mental disorder. In 2021, it was estimated to affect 357.43 million people globally, with a prevalence of 332.41 million cases and a burden of 56.33 million disability-adjusted life years [1]. It is a chronic and recurrent illness, with the risk of future episodes increasing with each recurrence, leading to a progressively worsening course of illness. Globally, depression is a leading cause of disability and imposes significant social and economic burdens [2,3]. Its impact extends beyond individuals to affect healthcare systems, workplaces, educational settings, and patient outcomes. In healthcare settings, depression contributes to misdiagnosis and reduced quality of service. Among workers, it leads to lost productivity, higher rates of absenteeism, and increased workplace accidents. Students with depression often experience declining academic performance and social difficulties, while patients with physical illnesses face higher mortality risks and poor treatment adherence [4]. The societal impact of depression spans across mental and physical health, social functioning, and economic stability, affecting individuals from adolescence through old age. The literature also highlights how factors such as sex, age, ethnicity, co-morbid conditions, and societal changes influence the prevalence and impact of depression [5]. The impact of depressive disorders is greatest among females and people aged 60 to 64 within their respective demographic groups [1]. Importantly, early prevention strategies—including family-based and individual interventions—can reduce the risk of developing depression by up to 21% [4]. Improving the cost-effectiveness of treatment, increasing awareness, and addressing social determinants of health are key to mitigating the burden of this widespread disorder.

Research demonstrates a complex and multifaceted relationship between chronic stress and major depressive disorder (MDD). Chronic stress induces dysregulation of the hypothalamic–pituitary–adrenal (HPA) axis, leading to excessive cortisol levels that damage hippocampal and prefrontal circuits involved in mood regulation. This pathway is a long-standing and well-supported contributor to depression’s development [6]. These neuroendocrine alterations are believed to underlie vulnerability to mood and anxiety disorders, and normalization of HPA axis function through psychopharmacologic or psychotherapeutic interventions may improve treatment outcomes.

Evidence suggests that chronic stress is significantly associated with the onset of MDD and may heighten sensitivity to acute stressors. Interestingly, chronic stress not only predicts major depressive episodes (MDEs) independently but also increases the likelihood of encountering acute stress events—a process known as stress generation. Acute stressors tend to have a stronger immediate association with MDE onset, but the sensitizing effect of chronic stress can amplify the impact of these acute stressors, contributing to depressive episodes [7]. The relationship between stress and depression extends beyond neuroendocrine mechanisms. Chronic stress has been linked to dysregulation in the immune, cardiovascular, and nervous systems, further connecting it to both anxiety and mood disorders. If left untreated, chronic stress may lead to significant impairments, including MDD and other serious health conditions such as cardiovascular disease [8].

Research into MDD has undergone a significant paradigm shift—from a traditional neuron-centric view to one that increasingly recognizes the critical role of astrocytes in the pathogenesis of depression. Astrocytic dysfunction is now considered a central factor in the neurobiology of MDD, with growing evidence from clinical, preclinical, and post-mortem studies demonstrating alterations in astrocyte density, morphology, and function [9,10]. Findings from both human patients and animal models show decreased astrocyte numbers, astroglia atrophy, and functional impairments. These changes are closely associated with depressive-like phenotypes in animals, suggesting that astrocyte pathology may play a causative role in MDD [10]. Moreover, current antidepressant therapies have been shown to exert beneficial effects on astrocytes, further supporting their involvement in mood regulation. Astrocytes perform essential regulatory functions in the brain, including neurotransmitter uptake and recycling, metabolic and trophic support to neurons, regulation of synaptic plasticity and maintenance of the blood–brain barrier. Disruption of these functions can contribute to the development of depressive symptoms. One key mechanism is neuroinflammation, in which astrocyte reactivity and release of pro-inflammatory cytokines promote synaptic dysfunction and cognitive impairment. These processes are influenced by psychosocial stress, aging, and peripheral immune activation [11]. Recent reviews synthesize findings on the multifaceted roles of astrocytes in MDD, particularly regarding their involvement in neuroplasticity, neurotransmission, immune signaling, and metabolic regulation. Consequently, targeting astrocyte-mediated pathways—including anti-inflammatory agents, metabolic modulators, and astrocyte-specific interventions—is being explored as a novel therapeutic strategy for depression [9,11].

A bibliometric analysis conducted by Lin et al. [12] reflects a growing global research interest in astrocyte-related mechanisms in depression. Analyzing 1.502 publications from 78 countries, the study identified key research hotspots, including synaptic plasticity, neuroinflammation, and molecular signaling. Influential authors and foundational works were highlighted, underscoring the rapid expansion of this emerging field [12].

These findings position astrocytes as crucial cellular hubs in the pathophysiology of depression. Their molecular complexity, involvement in key neurobiological functions, and responsiveness to stress make them promising therapeutic targets for the development of glia-focused antidepressant strategies.

## 2. Astrocytes in Brain Homeostasis and Mood Regulation

Astrocytes are multifunctional glial cells essential for maintaining central nervous system (CNS) homeostasis [13]. Far from being mere support cells, astrocytes actively contribute to synaptic signaling, metabolic regulation, and the maintenance of the neurovascular unit (NVU) and blood–brain barrier (BBB) [14]. Traditionally, classical neuroanatomy has divided astrocytes into two principal forms—protoplasmic and fibrous based on their morphology and localization [15]. Protoplasmic astrocytes, predominant in gray matter, display a star-like morphology with thick primary branches that further divide into numerous fine, highly ramified processes. In contrast, fibrous astrocytes are more abundant in white matter and are characterized by elongated, filamentous projections. This dichotomy, first described in early histological studies, has long served as the foundation for astrocyte classification [15]. However, advances in molecular and transcriptomic profiling have revealed that astrocyte diversity extends well beyond this binary distinction. Single-cell RNA transcriptomics has uncovered region-specific and functionally specialized astrocyte subpopulations, each expressing unique repertoires of neurotransmitter transporters, ion channels, and metabolic enzymes [16]. Such findings highlight that astrocyte heterogeneity is not only morphological but also deeply embedded at the molecular level, shaping their roles in synaptic regulation, metabolic support, and neurovascular interactions [17]. Single-cell transcriptomics has demonstrated that astrocytes vary across brain regions (e.g., hippocampus vs. cortex) and states (e.g., awake vs. asleep). Even within gray matter, astrocytes display diverse expression profiles of neurotransmitter transporters, ion channels, and metabolic enzymes, suggesting functional specialization. We can distinguish radial astrocytes—Müller glia in the retina, Bergmann glia in the cerebellum, velate astrocytes in the cerebellum and olfactory bulb, and human-specific astrocytes. Compared with rodent astrocytes, human astrocytes are larger, more complex, and form more extensive domains. These interlaminar astrocytes (found in cortical layer I) and varicose projection astrocytes are unique and may contribute to higher cognitive functions [18].

### 2.1. Astrocytes Neurotransmission Regulation

Astrocytes are increasingly recognized as active participants in higher-order brain functions, including learning, memory, and executive processes. Although traditionally regarded as supportive cells, they contribute to cognition through their regulation of synaptic activity, modulation of neuronal networks, and integration of metabolic and vascular signals. By coordinating neurotransmission, energy supply, and homeostasis, astrocytes provide a cellular basis for the plasticity and adaptability required for cognitive performance [18,19]. Astrocytes are the principal regulators of extracellular neurotransmitter concentrations in the CNS, ensuring both synaptic fidelity and neuronal survival. Their fine processes closely envelop synapses, enabling rapid detection, clearance, and recycling of neurotransmitters from the extracellular space [18]. The most extensively studied system is glutamate uptake, mediated predominantly by the astrocytic excitatory amino acid transporters EAAT1 (GLAST) and EAAT2 (GLT-1). By rapidly removing glutamate following synaptic release, astrocytes prevent excitotoxicity, maintain the temporal precision of excitatory transmission, and preserve the capacity for synaptic plasticity [20,21]. After uptake, glutamate is converted into glutamine by astrocytic glutamine synthetase and shuttled back to neurons, completing the glutamate–glutamine cycle and ensuring a continuous supply of neurotransmitter precursors.

Astrocytes also regulate GABAergic transmission. Through GABA transporters (GAT-1 and GAT-3), they clear GABA from the synaptic cleft, thus shaping inhibitory signaling and preventing excessive neuronal inhibition [22]. Similarly, astrocytic uptake of glycine and extracellular adenosine triphosphate (ATP) contributes to the fine-tuning of inhibitory and purinergic signaling [23].

### 2.2. Gliotransmission

In parallel, astrocytes actively release gliotransmitters, positioning them as modulators of synaptic activity rather than passive support cells. Gliotransmission is typically triggered by intracellular calcium signaling and occurs via vesicular exocytosis, hemichannels, or transporter-mediated pathways. Key gliotransmitters include glutamate, which activates extrasynaptic n-methyl-d-aspartate (NMDA) receptors and can promote long-term potentiation (LTP) or long-term depression (LTD), thereby contributing directly to learning and memory [24]. Similarly, D-serine, a co-agonist at NMDA receptors, is crucial for synaptic plasticity, particularly in hippocampal circuits [25]. Also, astrocytic ATP release and its subsequent extracellular degradation to adenosine modulate neuronal excitability, synchronizing circuit activity to support efficient information processing, regulating sleep homeostasis, and suppressing seizures [26] and GABA, released via Best1 channels, provides tonic inhibition and contributes to network stability in regions such as the hippocampus and thalamus [27]. Astrocytes express type 1 cannabinoid receptors (CB1R), which regulate intracellular Ca^2+^ signaling, synaptic plasticity, memory, and metabolic processes [28,29]. Activation of CB1R triggers Ca^2+^-dependent release of gliotransmitters, including D-serine, thereby enhancing dendritic NMDA receptor activation and modulates dendritic spike thresholds [30,31]. Recent studies [31] have demonstrated that astrocytes are recruited by neuronal endocannabinoid signaling to release D-serine, thereby facilitating activity-dependent astrocyte–neuron communication in the hippocampus. This reciprocal signaling occurs selectively at theta-frequency (~10 Hz) neuronal firing, suggesting frequency-specific astrocytic engagement. Furthermore, this positive feedback loop between astrocytes and neurons is essential for spatial learning and memory, as disrupting astrocytic Ca^2+^ dynamics or deleting CB1R in astrocytes impaired this crosstalk and resulted in memory deficits. Collectively, these findings indicate that astrocytes modulate dendritic computations and spatial learning through endocannabinoid receptor–mediated, Ca^2+^-dependent gliotransmission [31]. An essential role of astrocytes has been revealed in oxytocin (OT)—mediated neuromodulation, expanding the classical view that OT acts solely on neurons [32,33]. OT, synthesized in the hypothalamus and distributed via long-range axonal projections, regulates a range of behaviors including social interaction, pain, and anxiety [34]. In the central amygdala (CeA)—a key structure in emotional regulation, Wahis et al. identified a distinct population of astrocytes expressing oxytocin receptors (OTRs) [32]. These OTR-positive astrocytes exhibited larger and more complex morphologies and were more interconnected than OTR-negative astrocytes. Activation of OTRs, either by optogenetically evoked OT release or pharmacological stimulation with the selective agonist Threonine-4, Glycine-7 Oxytocin (TGOT), produced robust astrocytic Ca^2+^ transients. Selective deletion of the OTR gene in CeA astrocytes confirmed the functional expression of astrocytic OTRs, and electrophysiological recordings demonstrated that OTR activation in astrocytes significantly modulated CeA neuronal activity. This modulation was mediated, at least in part, by astrocytic D-serine release acting on neuronal NMDA receptors. Together, these findings show that OT signaling through astrocytic OTRs induces Ca^2+^-dependent gliotransmission, thereby shaping neuronal network activity within the CeA and contributing to the modulation of emotion-related behaviors [32,33].

### 2.3. Astrocyte’s Metabolism

By tightly coupling neurotransmitter uptake with gliotransmitter release, astrocytes maintain extracellular homeostasis while dynamically modulating synaptic strength and plasticity, thereby supporting learning and memory. Importantly, these processes are energetically sustained by astrocytic metabolic functions, such as glycogen storage and lactate shuttling, highlighting the seamless integration of synaptic and metabolic support in CNS physiology. The high energy demands of neuronal activity require tight coordination between neuronal firing and local energy supply. Astrocytes play a central role in this process by coupling synaptic activity with metabolic support, thereby sustaining neurotransmission and protecting neurons from metabolic stress. One of the most important contributions of astrocytes to brain energy metabolism is the astrocyte–neuron lactate shuttle (ANLS). During periods of intense synaptic activity, astrocytes take up glutamate through EAAT transporters, a process tightly linked to enhanced glycolysis. This results in the production of lactate, which is exported via monocarboxylate transporters (MCT1 and MCT4) and delivered to neurons, where it is converted into pyruvate and used in oxidative phosphorylation [35]. Lactate is now recognized not only as a fuel source but also as a signaling molecule that influences synaptic plasticity and memory formation [36]. Astrocytes also serve as the brain’s main glycogen reservoir, since neurons lack significant glycogen stores. Glycogen, primarily localized in protoplasmic astrocytes, provides a rapid, on-demand energy buffer during periods of high activity or hypoglycemia. Mobilization of glycogen-derived lactate has been shown to be essential for long-term memory consolidation and resistance to metabolic stress, highlighting glycogen-derived lactate as a necessary substrate for cognitive resilience [37]. Notably, data from experimental in vitro and in vivo animal models show that the neuromodulator adenosine mediates neuronal activity-dependent astrocytic metabolic stimulation by acting on astrocytic A2B receptors. Activation of A2B receptors results in the recruitment of the canonical cyclic adenosine 3′,5′-monophosphate (cAMP)—protein kinase A (PKA) signaling pathway and boosts astrocyte glucose metabolism, followed by the release of lactate. In a mouse model, knockdown of A2B receptor expression in astrocytes led to major changes in brain energy metabolism, weakened synaptic plasticity in the hippocampus, severely disrupted recognition memory and sleep, indicating cAMP signaling as a key player in fundamental brain functions [38]. Beyond carbohydrate metabolism, astrocytes play a crucial role in maintaining the extracellular microenvironment by regulating pH, ion concentrations, and osmolarity, ensuring the stability of synaptic signaling required for efficient cognitive performance. Neuronal depolarization during neurotransmission produces rapid fluctuations in extracellular potassium, sodium, calcium, chloride, and bicarbonate levels, as well as changes in pH and osmotic balance. Astrocytes, together with oligodendrocytes, buffer these alterations through mechanisms involving carbonic anhydrase and diverse ion channels. A key process is potassium spatial buffering, whereby astrocytes sequester excess extracellular K^+^ released during neuronal firing and redistribute it via their gap junction–coupled syncytium into regions of lower activity, cerebrospinal fluid (CSF), or blood. This mechanism highlights the essential role of astrocytic networks in stabilizing the neuronal environment and preserving excitability [18].

### 2.4. Neurovascular Unit and Blood–Brain Barrier

Astrocytes are integral to the NVU, where they link neuronal activity to vascular responses and maintain the functional integrity of the BBB. Their perivascular endfeet almost completely ensheath the cerebral vasculature, positioning astrocytes to coordinate communication between neurons, endothelial cells, and pericytes [14]. Astrocytes are key mediators of activity-dependent changes in cerebral blood flow. Synaptically released glutamate activates metabotropic glutamate receptors on astrocytes, leading to increases in intracellular calcium. These Ca^2+^ signals propagate to astrocytic endfeet, where they trigger the release of vasoactive messengers such as prostaglandin E_2_ (PGE_2_), epoxyeicosatrienoic acids (EETs), nitric oxide (NO), and potassium ions [39]. Depending on the local metabolic and oxygenation state, these molecules can induce either vasodilation or vasoconstriction, thereby optimizing cerebral perfusion in response to neuronal demand [40]. This mechanism ensures that energy substrates are efficiently delivered to regions of heightened synaptic activity. Astrocytic calcium waves and intercellular communication via gap junctions allow the formation of large-scale glial networks that modulate neuronal oscillations. These oscillations, particularly in the theta and gamma frequency ranges, are central to attention, working memory, and decision-making [41]. Astrocytic endfeet also play a vital role in establishing and maintaining the BBB. While endothelial cells and their tight junctions constitute the physical barrier, astrocytes secrete a variety of factors—including angiopoietin-1, glial-derived neurotrophic factor (GDNF), and sonic hedgehog—that enhance tight junction integrity and support endothelial differentiation [42]. Additionally, aquaporin-4 (AQP4), which is highly enriched in astrocytic endfeet, regulates water transport and contributes to the clearance of interstitial solutes through the glymphatic system, a process critical for the removal of metabolic waste products such as amyloid-β [43]. Thus, astrocytes ensure both barrier stability and brain homeostasis.

### 2.5. Astrocytic Neurotrophic Support

**Figure 1:**
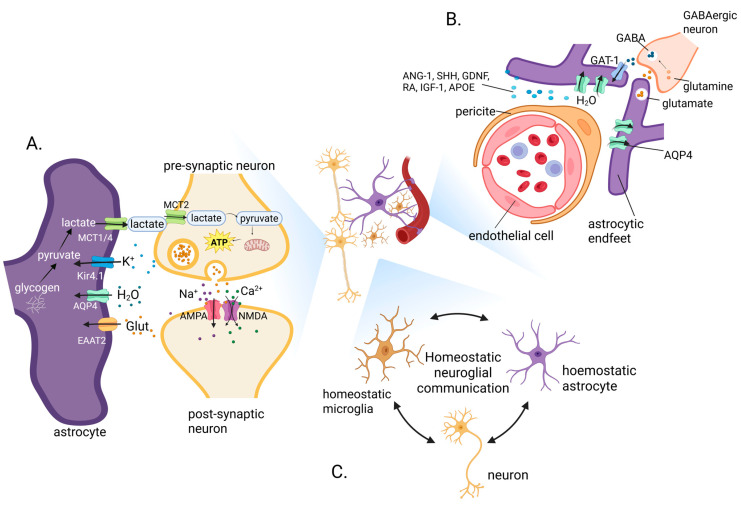
Astrocytic integration of synaptic, metabolic, and vascular homeostasis in the healthy brain. Astrocytes orchestrate brain homeostasis by coupling neurotransmission, energy metabolism, and neurovascular regulation. Astrocyte–neuron interactions at excitatory synapses involve glutamate uptake via EAAT2 and lactate transfer through the astrocyte–neuron lactate shuttle (ANLS), sustaining synaptic activity and preventing excitotoxicity (**A**). Astrocytic endfeet regulate water and ion balance via aquaporin-4 (AQP4) and maintain the BBB through the release of trophic and stabilizing factors such as angiopoietin-1 (ANG-1), sonic hedgehog (SHH), glial-derived neurotrophic factor (GDNF), retinoid acid (RA), insulin-like growth factor 1 (IGF-1), and Apolipoprotein E (APOE), while participating in the glutamate–glutamine–GABA cycle to support inhibitory signaling (**B**). Coordinated communication among astrocytes, neurons and microglia ensures homeostatic neuroglial signaling, integrating synaptic, immune, and metabolic functions essential for CNS stability and plasticity (**C**). Created in BioRender. Tomczak, J. (2025) https://BioRender.com/vruhbw4 (accessed on 27 October 2025).

Astrocytes play a fundamental role in maintaining neuronal health and plasticity through the synthesis and release of neurotrophic factors, which are essential for synaptic development, neuronal survival, and adaptive responses to experience. These trophic interactions not only sustain the structural integrity of neural circuits but also dynamically influence emotional regulation and cognitive performance. Astrocytes secrete a variety of growth factors, including brain-derived neurotrophic factor (BDNF), GDNF, nerve growth factor (NGF), and ciliary neurotrophic factor (CNTF) [44]. These molecules act on neuronal receptors to promote dendritic growth, synaptogenesis, and neuroprotection. BDNF, in particular, plays a key role in learning, memory, and emotional adaptation [45]. Astrocyte-derived BDNF enhances synaptic plasticity in the hippocampus and prefrontal cortex (PFC), regions critical for cognition and mood regulation [45]. Within emotion-regulating circuits such as the amygdala, hippocampus, and PFC, astrocytic release of BDNF and GDNF modulates synaptic efficacy and resilience to stress. For a detailed discussion of astrocytes and BDNF, see [45]. Astrocytic trophic factors also play critical roles in learning and memory. In the cortex, astrocyte-derived BDNF supports long-term potentiation and memory consolidation [46], while GDNF and CNTF contribute to synaptic maintenance and neuronal survival during aging or neurodegeneration [47]. Moreover, astrocytic secretion of thrombospondins (TSP-1 and TSP-2) facilitates excitatory synapse formation, supporting structural plasticity that underlies cognitive adaptability [48]. Different astrocyte subtypes contribute variably to neurotrophic support, e.g., protoplasmic astrocytes in gray matter release high levels of BDNF [49], directly influencing synaptic plasticity and emotional resilience, whereas fibrous astrocytes provide structural and trophic support to axons in white matter, maintaining myelin integrity, which is necessary for efficient cognitive processing [50]. Astrocytic neurotrophic support serves as a unifying mechanism linking cellular metabolism, synaptic plasticity, and behavioral adaptation. By modulating neuronal survival, dendritic complexity, and synapse formation, astrocyte-derived trophic factors underpin the structural basis of emotional stability and cognitive performance. Figure 1 illustrates astrocytic integration of synaptic, metabolic, and vascular homeostasis in the healthy brain.

### 2.6. Astrocyte Antioxidant Mechanisms and Their Implications for Cognitive, Emotional, and Learning Functions

In recent years, increasing evidence has highlighted the critical role of astrocytes in maintaining redox balance and mitigating oxidative stress within the brain. Through glutathione metabolism, enzymatic detoxification, and Nrf2-mediated gene regulation, astrocytes safeguard neurons against oxidative stress and maintain conditions essential for synaptic plasticity and network stability. Importantly, disturbances in astrocytic antioxidant capacity have been linked to impairments in cognition, emotion regulation, and learning processes—underscoring the functional interdependence between redox homeostasis and higher-order brain functions [51]. Astrocytes are the primary producers of glutathione (GSH) in the brain, maintaining intracellular concentrations of 2.8 µM—considerably higher than those found in neurons [52]. Through the γ-glutamyl cycle, astrocytes synthesize GSH and release it or its essential amino acids (cysteine, glutamate, glycine) into the extracellular space. Neurons, which have limited cystine uptake capacity, depend on these astrocyte-derived substrates to sustain their own GSH pools [53]. This astrocyte–neuron coupling forms the biochemical foundation for neuronal antioxidant defense. Astrocytes contribute to neuronal metabolism not only via lactate shuttling but also by providing reducing equivalents (NADPH) necessary for GSH regeneration, supporting neuronal energy metabolism and cognitive resilience under stress conditions [54]. Moreover, impaired astrocytic antioxidant metabolism, as observed during aging or metabolic dysfunction, correlates with cognitive decline and memory deficits [55].

Another component of redox homeostasis is the transcription factor nuclear factor erythroid 2–related factor 2 (Nrf2), a master regulator of the antioxidant response [56]. In astrocytes, Nrf2 activation induces the expression of GSH-related enzymes and phase II detoxifying genes. Importantly, astrocytes express high levels of antioxidant enzymes, including superoxide dismutases (SOD1/2), catalase, glutathione peroxidase (GPx), and glutathione-S-transferases (GSTs). These enzymes neutralize reactive oxygen species (ROS) such as superoxide and hydrogen peroxide, thereby maintaining local redox balance [57]. This positions Nrf2 as a central node linking glial redox signaling to neuronal survival and neuroprotection. Activation of astrocytic Nrf2 enhances resilience to chronic stress and depressive-like behaviors by maintaining neuronal integrity and regulating neuroinflammation [56]. Pharmacological Nrf2 activators, such as sulforaphane, improve cognitive and emotional outcomes in rodent models, likely through astrocyte-mediated antioxidant enhancement.

ROS at low levels can modulate signaling cascades that promote LTP, but excessive ROS disrupts NMDA receptor-mediated calcium homeostasis, and synaptic integrity [58]. Astrocyte-derived GSH protects neurons from such oxidative disruptions. Experimental depletion of GSH impairs hippocampal LTP and spatial learning, whereas GSH supplementation restores these functions [59,60]. Hence, astrocytic antioxidant support is indispensable for maintaining redox conditions conducive to synaptic plasticity. Emotion-related brain regions such as the PFC, amygdala, and hippocampus exhibit high oxidative metabolism and are thus vulnerable to oxidative stress. Astrocyte antioxidant systems buffer these regions against ROS accumulation during stress or emotional arousal. Preclinical models indicate that redox imbalance in astrocytes contributes to stress-induced behavioral alterations and mood disorders [61].

## 3. Molecular Mechanisms Linking Chronic Stress to Astrocyte Dysfunction in the Pathophysiology of Depression

### 3.1. Astrocytic Glucocorticoid Dysregulation

Sustained activation of the HPA axis results in prolonged elevation of circulating glucocorticoids, which, while adaptive in acute situations, become detrimental when persistently elevated [62,63]. Prolonged glucocorticoid signaling desensitizes glucocorticoid receptors (GRs) in limbic and cortical regions, disrupts negative feedback control, and promotes allostatic overload [64]. Under chronic stress conditions, glucocorticoid activation of GRs initiates extensive transcriptional reprogramming in astrocytes, with significant downstream impacts on cellular structure, metabolic function, and synaptic support.

Experimental blockade of gap junctions induces depressive-like behavior, while antidepressants and glucocorticoid receptor antagonists restore connexin expression and behavioral function, highlighting astrocytic gap junctions as mechanistic links between stress and mood regulation [65,66]. Moreover, astrocytic GR signaling is a sensitive interface between systemic stress and cellular dysfunction. Reduced GR expression in astrocytes has been observed in both stressed animals and postmortem brain tissue from individuals with MDD, correlating with decreased ATP release and neuronal support. Conditional deletion of astrocytic GRs disrupts glucose metabolism and aversive learning, while restoration of GRs in the mPFC normalizes both molecular markers and behavioral outcomes, providing strong evidence of their role in resilience [67,68]. Complementary studies in human iPSC-derived astrocytes demonstrate that chronic, but not acute, cortisol exposure induces transcriptional reprogramming affecting GPCR signaling, ion homeostasis, and synaptic regulation. These effects are most pronounced in cells derived from MDD patients, suggesting that chronic stress imprints a disease-specific molecular phenotype in astrocytes [69].

Equally important, glucocorticoids disrupt astrocyte-neuron metabolic coupling by impairing the astrocyte lactate shuttle—an indispensable pathway through which astrocytes provide energy-rich lactate to neurons. A compromised failing lactate shuttle can contribute to neuronal energy deficits, compromising synaptic plasticity and cognitive function over time [70]. Complicating the picture, chronic glucocorticoid exposure also suppresses the expression of GRs themselves in astrocytes, establishing a deleterious, self-amplifying feedback loop. This begins with the induction of serum- and glucocorticoid-regulated kinase 1 (SGK1), which phosphorylates and suppresses the activity of the transcription factor Forkhead box O3a (FOXO3a) [71,72]. FOXO3a normally suppresses the expression of liver kinase B1 (LKB1), the pivotal upstream activator of the energy sensor AMP-activated protein kinase (AMPK). Impaired activation of AMPK not only disrupts energy homeostasis but also enables constitutive activity of histone deacetylase 5 (HDAC5), leading to repressive histone modifications at the GR gene promoter and further downregulation of GR expression. Thus unfolds a molecular cascade—from GR downregulation to epigenetic remodeling—that entrenches astrocytic dysfunction [73].

Glucocorticoid signaling also exerts direct effects on astrocytic function. Lu et al. reported that deletion of the astrocyte-specific GR gene (NR3C1) induced social avoidance and impaired ATP release via downregulation of the PI3K–AKT signaling pathway, which is essential for cell survival and stress adaptation [67]. Translational studies have further shown that individuals with a history of early-life adversity exhibit NR3C1 hypermethylation, contributing to a dysregulated HPA axis response to stress [74,75]. Preclinical research has laid the foundation for understanding the interplay between chronic stress, neuroinflammation, and depressive phenotypes. One of the central mechanisms involves glucocorticoid-induced downregulation of critical astrocytic proteins, including GFAP [65] and pivotal glutamate transporters such as GLT-1 [76]. These proteins are essential for maintaining the integrity of the astrocytic network, the fast clearance of glutamate from the synaptic cleft, and overall glutamate homeostasis. Deficiencies in GFAP undermine astrocyte structural stability. Downregulation of GLT-1 causes glutamate accumulation, which heightens the risk of excitotoxicity by prolonging the presence of extracellular glutamate following neurotransmission [77]. This leads to overstimulation of NMDA receptors, calcium overload, and astrocytic apoptosis—particularly in the hippocampus. In rodent models, 21 days of chronic unpredictable mild stress (CUMS) elicited marked depressive-like behaviors and significantly increased hippocampal glutamate levels, along with altered expression of proteins related to apoptosis and signaling pathways [78]. Emerging evidence highlights the crucial role of astrocytes in regulating glutamatergic signaling, and how this role is compromised under stress. Understanding these glia-neuron interactions offers promising avenues for the development of more effective treatments for stress-related disorders, including depression [79].

The hippocampus, particularly vulnerable due to its dense GR population, exhibits Cornu Ammonis area 3 (CA3) dendritic retraction, spine loss, and suppressed neurogenesis in the dentate gyrus, which collectively disrupt contextual learning, pattern separation, and inhibitory feedback to the HPA axis [80].

Moreover, glucocorticoid signaling regulates astrocytic BDNF, a key neurotrophic factor essential for neuronal survival and synaptic function. In MDD, this dysregulation contributes to reduced BDNF levels, particularly in the hippocampus and PFC—regions commonly affected in the disorder. The resulting decline in BDNF may underlie the structural and functional brain alterations observed in MDD, emphasizing the crucial role of astrocytic BDNF in stress-related neuropathology. Conversely, reduced astrocytic BDNF expression has been observed in models of depression and chronic stress, further linking impaired glial trophic support to the development of affective disorders [81].

### 3.2. Neuroinflammatory Pathways Linking Chronic Stress to Depressive Pathophysiology

Chronic stress fosters a systemic pro-inflammatory milieu characterized by elevated interleukin-1β (IL-1β), interleukin-6 (IL-6), and tumor necrosis factor-α (TNF-α). These cytokines have been consistently associated with increased depressive symptom severity, treatment resistance, and poor prognosis [82]. They can cross the BBB or signal indirectly through vascular and neural pathways, initiating neuroinflammatory cascades that involve both microglia and astrocytes. Experimental evidence shows that cytokine exposure alters neurotransmitter metabolism, reduces synaptic plasticity, and directly influences neurogenesis, as exemplified by IL-1β-mediated suppression of hippocampal cell proliferation and IL-6 or TNF-α–induced impairments in glutamatergic signaling [83]. At the neuroanatomical level, chronic stress exerts profound and region-specific remodeling of circuits essential for cognitive and emotional regulation. Preclinical research has further laid the foundation for understanding the interplay between chronic stress, neuroinflammation, and depressive phenotypes. Various rodent models of stress exposure—including chronic mild stress (CMS), learned helplessness, and repeated social defeat stress—consistently induce a melancholic-like behavioral profile, insulin resistance, and elevation of peripheral pro-inflammatory cytokines such as IL-1β, IL-6, and TNF-α [84]. Notably, IL-6 has emerged as a key mediator of stress vulnerability. In a study by Hodes et al., IL-6 levels were found to be up to 27-fold higher in stress-susceptible mice compared to resilient counterparts following repeated social defeat. Administering an IL-6 monoclonal antibody prior to stress exposure prevented the development of social avoidance, implicating IL-6 as a causal factor in stress susceptibility [85]. Other studies have demonstrated a critical interaction between inflammatory signaling and glucocorticoid dynamics in the brain. Stress-susceptible mice have shown elevated hippocampal IL-1β, increased adrenal activity, and reduced hippocampal neurogenesis. Interestingly, adrenalectomy abolished depressive-like behaviors, while corticosterone replacement restored them—even in mice lacking IL-1 receptors—suggesting that adrenocortical activation modulates the relationship between inflammation and depression [82].

Astrocytes are central to the brain’s immune and metabolic responses, particularly under chronic stress conditions. Two key inflammatory signaling pathways—NF-κB and signal transducer and activator of transcription 3 (STAT3)—play integral roles in regulating astrocyte reactivity. Activation of NF-κB stimulates IL-6 release, which in turn activates STAT3, initiating gene expression profiles that promote astrogliosis and glial scar formation. These pathways are crucial for mediating CNS responses to injury, including spinal cord injury (SCI), and have become therapeutic targets for modulating astrocyte function in neurodegeneration and stress-related disorders [76]. The spatial and temporal activation of NF-κB and STAT3 following injury supports their role in early astrocyte reactivity. These pathways are rapidly activated in regions with severe tissue damage and blood–brain barrier disruption and remain upregulated in chronic gliotic lesions [86]. Moreover, STAT3 drives distinct inflammatory transcriptional signatures in astrocytes, some of which are promoted and others inhibited by its activation. These signatures are conserved across experimental models and are observed in human brains affected by Alzheimer’s disease and hypoxic–ischemic encephalopathy [87]. Within the CNS, stress-induced inflammation is exacerbated by increased permeability of the BBB. Animal studies have demonstrated stress-mediated reductions in tight junction proteins such as claudin-5 (Cldn5), occludin, and zonula occludens-1 (ZO-1)—particularly in key mood- and reward-related brain regions like the nucleus accumbens, hippocampus, and PFC—facilitating peripheral cytokine infiltration. Chronic administration of the tricyclic antidepressant imipramine restores tight junction protein expression and attenuates depression-like behaviors in these models. Intriguingly, commonly used psychotropic drugs like lithium, haloperidol, and chlorpromazine also enhance Cldn5 levels in brain endothelial cells, suggesting that their clinical effectiveness may partly stem from the restoration of BBB integrity [88].

### 3.3. Mitochondrial Vulnerability and Epigenetic Reprogramming as Convergent Mechanisms in Stress-Related Brain Pathophysiology

Mitochondrial dysfunction and oxidative stress are pivotal contributors to astrocytic and neuronal damage under pathological conditions. Astrocytes, which play a critical role in maintaining neuronal health and homeostasis, undergo significant mitochondrial alterations in response to chronic stress. These alterations include changes in mitochondrial membrane potential and elevated production of ROS, which disrupt cellular energy metabolism and promote cell injury. Mitochondria are central to both normal physiological processes and cell death pathways, particularly in astrocytes that support neuronal function. Notably, astrocytic and neuronal mitochondria respond differently to stressors such as oxygen-glucose deprivation, with astrocytes often exhibiting greater resilience. This differential response is tightly regulated by the Bcl-2 family of proteins, which govern mitochondrial-mediated apoptosis and other forms of cell death [89]. Disruption of the STAT3 signaling pathway in astrocytes further exacerbates oxidative damage. The absence of STAT3 activity leads to increased ROS production, reduced glutathione levels, impaired mitochondrial function, and a decline in both mitochondrial membrane potential and ATP synthesis. These alterations culminate in decreased astrocyte proliferation and impaired neural support [90]. Mitochondrial ROS can also induce calcium release from the endoplasmic reticulum (ER), promoting mitochondrial calcium overload and the opening of the mitochondrial permeability transition pore (mPTP). This event is a critical trigger for necrotic cell death. While transient mitochondrial depolarizations may be reversible, sustained mPTP opening leads to irreversible mitochondrial collapse and astrocyte demise [91]. Following brain injury, oxidative stress intensifies mitochondrial dysfunction in both astrocytes and neurons. Mechanisms include protein oxidation, mPTP opening, and depletion of mitochondrial NAD(H), a coenzyme essential for oxidative phosphorylation. Notably, reoxygenation under hyperoxic conditions significantly worsens mitochondrial impairment and accelerates cell death, especially in neurons [92]. Collectively, these findings underscore the intricate relationship between oxidative stress and mitochondrial dysfunction in astrocytes, highlighting their crucial role in the pathophysiology of neurodegeneration and stress-related neuropathologies.

**Figure 2:**
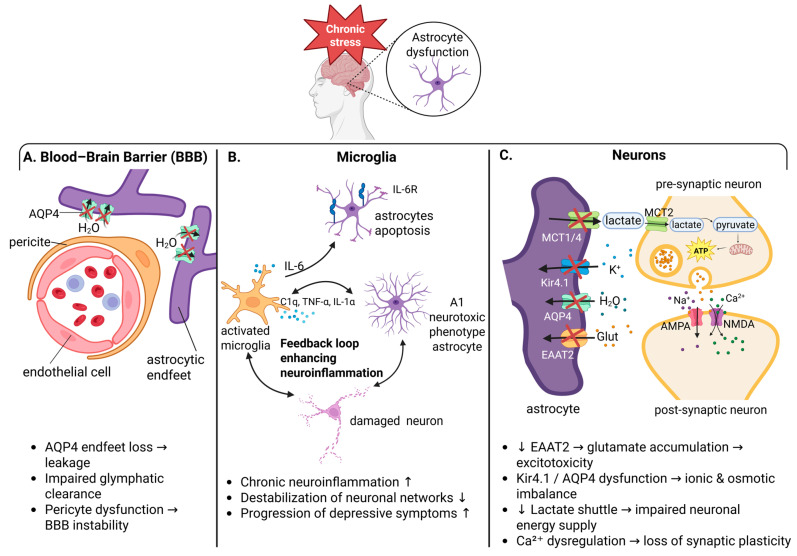
Schematic representation of astrocytic dysfunction under chronic stress and its contribution to the pathophysiology of major depressive disorder (MDD). Prolonged glucocorticoid exposure and systemic inflammation drive structural and molecular impairments in astrocytes, which in turn destabilize multiple levels of brain homeostasis. (**A**) At the neurovascular interface, loss of aquaporin-4 (AQP4) polarization in astrocytic endfeet and pericyte dysfunction compromise blood–brain barrier (BBB) integrity and glymphatic clearance. (**B**) Activated microglia release interleukin-6 (IL-6), interleukin-1α (IL-1α), tumor necrosis factor-α (TNF-α), and complement component 1q (C1q), promoting astrocytic apoptosis and induction of A1 neurotoxic astrocytes. This maladaptive glia–glia crosstalk creates a feedback loop that sustains neuroinflammation and contributes to neuronal injury. (**C**) At the tripartite synapse, reduced EAAT2-mediated glutamate uptake and impaired Kir4.1/AQP4-dependent ionic buffering induce excitotoxic stress. In parallel, loss of astrocytic metabolic support via the lactate shuttle and dysregulated Ca^2+^ signaling further impair neuronal plasticity and resilience. Collectively, these mechanisms highlight astrocytes as central mediators linking chronic stress to synaptic dysfunction, neuroinflammation, and depressive symptomatology. Created in BioRender. Tomczak, J. (2025) https://BioRender.com/k4ub4bm (accessed on 27 October 2025).

Parallel to these metabolic and mitochondrial vulnerabilities, chronic stress exerts powerful epigenetic effects that shape long-term neural function. Epigenetic mechanisms such as histone modifications, DNA methylation, and the regulation of gene expression by non-coding RNAs, including microRNAs (miRNAs) play a pivotal role. Prolonged stress exposure induces persistent changes in neural circuits that underlie mood and cognitive processes. These changes are often driven by alterations in gene expression that do not involve changes to the DNA sequence itself. Instead, stress triggers epigenetic reprogramming, which can cause long-lasting effects on brain function. Both animal models and clinical studies support a critical role for epigenetic dysregulation in stress-related psychiatric disorders [93]. One particularly important epigenetic regulator in this context is microRNA-124 (miR-124), which plays a central role in modulating stress resilience. Chronic stress significantly reduces the expression of miR-124 in the hippocampus, a brain region critical for mood regulation. Experimental overexpression of miR-124 in hippocampal neurons has been shown to confer resilience to stress-induced depression-like behaviors, whereas its inhibition increases vulnerability. Mechanistically, miR-124 regulates key targets involved in stress responses, including histone deacetylases HDAC4 and HDAC5, as well as glycogen synthase kinase 3 beta (GSK3β). Dysregulation of these proteins contributes to structural and functional neuronal changes, including dendritic remodeling in the dentate gyrus, a hallmark of stress-induced neural plasticity alterations [94]. At the systems level, the limbic–hypothalamic–pituitary–adrenal (LHPA) axis is a central regulator of the physiological stress response. Psychogenic stressors can induce genome-wide epigenetic changes, particularly in DNA methylation patterns within the CNS. These changes have been observed both in animal models and in human studies, pointing to their importance in stress adaptation and maladaptation [95]. Astrocytes have emerged as pivotal cellular targets of chronic stress, showing both structural and functional abnormalities in brain regions particularly vulnerable to depression (Figure 2).

## 4. Astrocyte-Specific Gene Expression Changes in Depression

### 4.1. Astrocytic Dysregulation of Glutamatergic, GABAergic, and Purinergic Signaling in Major Depressive Disorder

Postmortem and neuroimaging studies consistently reveal profound impairments in the glutamate–GABA–glutamine cycle in individuals with MDD, particularly within frontolimbic regions such as the PFC and hippocampus. The functional consequences of astrocytic dysfunction are profound and multifaceted. Impaired EAAT1/2 function reduces glutamate clearance, while deficits in inward-rectifier potassium channels and AQP4 impair ionic and osmotic buffering, creating an excitotoxic environment that destabilizes neural networks [96]. These disruptions are marked by reduced expression of astrocytic glutamate transporters EAAT2 and EAAT1 (encoded by SLC1A2 and SLC1A3, respectively), as well as glutamine synthetase (GS), the enzyme responsible for converting glutamate to glutamine. Correspondingly, neuroimaging studies have reported lower glutamate and glutamine levels in these regions, reinforcing the notion of astrocytic dysfunction in MDD [97,98]. Parallel disruptions are evident in the locus coeruleus (LC), a brainstem hub of noradrenergic signaling, where astrocytes similarly exhibit reduced expression of SLC1A2 and SLC1A3, suggesting impaired glutamate clearance. Animal models of depression, including those using CMS, chronic unpredictable stress (CUS), corticosteroid exposure, or congenital helplessness, replicate these changes, showing reduced mRNA and protein levels of GLT-1 and GLAST across key brain regions. Corticosterone-treated mice display increased glutamate/D-serine release [99], while in flinders sensitive line (FSL) rats, GLAST downregulation impairs glutamate reuptake, leading to enhanced glutamatergic transmission in the hippocampal CA1 region [100]. Similarly, knockdown of GLT-1 and GLAST in the infralimbic cortex enhances gliotransmission and induces depression-like phenotypes [101]. In the chronic restraint stress model, increased astrocytic connexin-43 hemichannel activity in the ventral hippocampus drives excessive glutamate and D-/L-serine release, overactivating NMDA receptors and triggering depressive behaviors. Pharmacological blockade of connexin-43 hemichannels prevents these effects and normalizes extracellular glutamate levels [102].

In addition to glutamatergic dysfunction, GABAergic signaling is also markedly impaired in MDD. Imaging studies show reduced cortical GABA levels, while postmortem analyses reveal altered expression of GABA_A_ receptor subunits—including β3, δ, and γ2 in the dorsolateral PFC, and α1 and β3 in the anterior cingulate cortex (ACC) of suicidal individuals [103]. Chronic stress paradigms in rodents similarly result in decreased hippocampal GABA content, impaired GAB_AB_ receptor function, and reduced synaptic inhibition, which together drive hyperexcitability of pyramidal neurons [104]. Early-life stress compounds this effect, downregulating GABA_A_ receptor subunits in limbic areas and correlating with behavioral manifestations of depression and anxiety [105]. Astrocytes play a central role in regulating GABA metabolism, not only through uptake and synthesis but also via release mechanisms. In FSL rats, reactive astrocytes display increased monoamine oxidase-B (MAO-B) activity, leading to excessive GABA release and heightened tonic inhibition via extrasynaptic GABA_A_ receptors. This abnormal inhibition impairs synaptic plasticity, particularly in the PFC. Importantly, pharmacological blockade of astrocytic calcium signaling or MAO-B activity has been shown to restore plasticity, though the extent to which such interventions alleviate depressive symptoms remains under investigation [106].

Beyond their roles in glutamate and GABA homeostasis, astrocytes also shape neuronal excitability and synaptic function through gliotransmission—the Ca^2+^-dependent release of neuroactive substances such as glutamate, ATP, adenosine, and D-serine [107]. Astrocytes express receptors and transporters for various neurotransmitters and neuromodulators, including glutamate, GABA, adenosine, noradrenaline, serotonin, acetylcholine, and endocannabinoids [99,108]. Dysregulation of these receptor-mediated signaling pathways has emerged as a key contributor to MDD.

Purinergic signaling has received particular attention. Imaging studies report reduced ATP levels in frontal cortical regions of MDD patients [109], while postmortem findings in suicide subjects show decreased expression of ENTPD2, an astrocyte-enriched enzyme responsible for hydrolyzing ATP to ADP and AMP [110]. Rodent models support these findings. CSDS mice display reduced ATP levels in the PFC and hippocampus, while boosting astrocytic purinergic signaling in Itpr2^−^/^−^ mice induces antidepressant effects via P2X2 receptor activation. Similarly, astrocyte-specific deficits in ATP release (e.g., in Itpr2^−^/^−^ or dn-SNARE mice) can be rescued by systemic ATP administration [111]. In parallel, astrocytic insulin receptor deficiency increases anxiety and depressive-like behaviors, attributed to impaired purinergic signaling from astrocytes to dopaminergic neurons [112]. In the mPFC, disrupted EET signaling impairs ATP release from astrocytes, contributing to depressive phenotypes [113]. Moreover, the absence of GRs in astrocytes has been associated with depression-like behavior due to reduced ATP release and suppressed Ca^2+^ responses to stress [67]. ATP can also be released through non-exocytotic pathways, which appear altered in depression. For instance, in the CUS model, connexin-43 and Panx-1 channels, both permeable to ATP, show reduced expression in the PFC [114]. Blocking these channels in healthy animals induces depressive-like behavior, highlighting their role in astrocyte-mediated ATP signaling [115]. Adenosine, a breakdown product of ATP, is another critical purinergic signal in depression, released by both astrocytes and neurons [38]. Elevating astrocyte-derived adenosine and activating adenosine A1 receptors produce robust antidepressant effects in animal models [116]. Non-pharmacological treatments such as sleep deprivation or deep brain stimulation of the infralimbic PFC also depend on intact astrocyte-neuron communication, particularly adenosine A1 receptor signaling [117].

### 4.2. Astrocytic Markers, Epigenetic Alterations, and Structural Network Dysfunction

Epigenetic studies further corroborate the role of astrocytes in MDD. Genome-wide DNA methylation analyses have identified differentially methylated regions in astrocytic markers, with particular emphasis on genes such as glutamate ionotropic receptor kainate type subunit 2 (GRIK2) and brain-enriched guanylate kinase-associated protein (BEGAIN). These regions displayed significantly reduced methylation in individuals with depression, correlating with altered gene expression. Importantly, methylation changes in BEGAIN were primarily driven by non-neuronal cells—likely astrocytes—and were shown to affect gene expression in vitro, suggesting a mechanistic link between astrocyte epigenetics and depressive pathology [118]. In the hippocampus, the transcription factor TCF7L2 has been identified as a potential key regulator of astrocyte function in depression. Its expression is reduced in mouse models of depression but restored following amitriptyline treatment. TCF7L2 regulates astrocyte proliferation, differentiation, and cytokine signaling, indicating a role in both stress adaptation and antidepressant efficacy [119].

Further supporting astrocytic compromise in MDD, decreased expression of GFAP—a structural and functional astrocyte marker—has also been observed in the LC [120]. Moreover, S100β, a calcium-binding protein predominantly expressed by astrocytes, shows elevated levels in the cerebrospinal fluid and serum of patients with MDD. These increased peripheral levels are thought to reflect either astrocytic injury or active secretion in response to pathological processes. Paradoxically, within brain tissue itself, both S100β expression and astrocyte density are reduced, suggesting a depletion or dysfunction of the astrocytic population at the cellular level. This dissociation between peripheral elevation and central depletion further emphasizes the complexity of astrocytic involvement in MDD and underscores the importance of considering both central and peripheral astrocyte-derived markers when investigating the neurobiology of depression [82].

Astrocytic communication is compromised by stress-induced downregulation of connexin-43 and connexin-30, weakening gap-junction coupling and disrupting syncytial networks. This uncoupling reduces astrocytic ability to distribute metabolites and ions across large networks, impairing neuronal support [121]. Experimental blockade of gap junctions induces depressive-like behavior, while antidepressants and glucocorticoid receptor antagonists restore connexin expression and behavioral function, highlighting astrocytic gap junctions as mechanistic links between stress and mood regulation [65,66]. Postmortem studies have shown reduced expression of connexin-43 and connexin-30 proteins and their mRNAs in key mood-regulating regions, including the PFC, orbitofrontal cortex, hippocampus, LC, mediodorsal thalamus, and caudate nucleus. These alterations have been observed in both MDD patients and animal models of depression [122,123].

### 4.3. AQP4 and Water Homeostasis in Depression

Astrocytic water homeostasis, closely linked to gap junction functionality, is largely governed by AQP4 [124]. AQP4 is a water channel protein highly enriched in astrocytic endfeet surrounding cerebral blood vessels. It plays a crucial role in regulating water and ion exchange, astrocyte process extension, glutamatergic signaling, and BBB integrity [62]. In depression, AQP4 dysfunction has been implicated in both clinical and preclinical studies. Postmortem analyses have revealed reduced AQP4 expression and astrocytic endfoot coverage of blood vessels in the PFC gray matter [125], as well as in the hippocampus [122] and LC. Similarly, animal models of depression—such as CMS, chronic corticosterone treatment, and stress-sensitive rat strains—show decreased AQP4 protein levels in multiple brain regions, including the PFC, hippocampus, LC, and choroid plexus. High-anxiety behavior rats also exhibit reduced AQP4-positive astrocyte coverage in the adult PFC [62,126]. AQP4 knockout (KO) mice, which model astrocytic dysfunction, present with cognitive deficits, impaired synaptic plasticity, and increased susceptibility to stress-induced depressive behaviors [127,128].

Moreover, AQP4 deficiency impairs potassium buffering, resulting in slower K^+^ clearance, heightened extracellular K^+^ levels, and neuronal hyperexcitability [129]. These mice also display reduced expression of synapsin-1 and GDNF and show decreased hippocampal neurogenesis, processes essential for mood regulation. Consistently, AQP4 KO animals exhibit deficits in LTP and altered neurotrophin signaling, which correlate with learning and memory impairments [127,130]. Notably, AQP4 signaling is highly sensitive to neuroinflammatory states. In vitro studies show that AQP4-deficient astrocytes have an attenuated cytokine response, producing lower levels of TNF-α and IL-6 following lipopolysaccharide (LPS) stimulation. Pericytes normally regulate AQP4 polarization, and their dysfunction under stress further exacerbates BBB instability [81]. In vivo CUS exposure leads to reduced AQP4 polarization, enhanced oxidative stress, and elevated neuroinflammation. Importantly, impaired AQP4 function also disrupts glymphatic clearance, allowing accumulation of inflammatory mediators and oxidative damage in the brain. While the exact mechanisms are still being investigated, these findings strongly suggest that AQP4 dysfunction contributes to depressive pathology, identifying it as a potential therapeutic target [9,131,132].

### 4.4. Astrocytic Metabolic and Calcium Signaling Dysregulation in MDD

Astrocytic metabolic dysfunction adds another layer of pathology. The astrocyte-to-neuron lactate shuttle, essential for sustaining synaptic potentiation, is impaired under chronic stress, reducing neuronal energy supply. Restoration of lactate transfer rescues long-term potentiation and alleviates depressive-like behaviors, underscoring the role of astrocytic metabolic failure in stress-induced deficits [133]. Beyond metabolic disruption, inflammation is a well-documented contributor to MDD pathophysiology [134]. Elevated levels of pro-inflammatory cytokines—including IL-1β, IL-6, TNF-α, and C-reactive protein—have been reported in MDD patients [135]. Astrocytes, due to their ability to both produce and respond to cytokines, and their close interaction with microglia, are increasingly recognized as key players in neuroinflammatory processes associated with depression [136]. Microglia-driven induction of A1-like astrocytes inhibits neuronal activity in prefrontal and hippocampal circuits, creating a vicious cycle of glial-neuronal toxicity [11,137,138]. Astrocytic Ca^2+^ signaling is also crucial during neurodevelopment. For instance, dampening cortical astrocytic Ca^2+^ activity in early development—achieved by overexpressing plasma membrane calcium-transporting ATPase 2 (PMCA2)—leads to depressive-like behaviors, impaired social interaction, and synaptic abnormalities in adulthood [139]. These deficits are reversed by chemogenetic reactivation of astrocytic Ca^2+^ signaling, highlighting its essential role in neural circuit formation and long-term behavioral outcomes. Similarly, IP3 receptor type 2 knockout (Itpr2^−^/^−^) mice, which exhibit near-complete loss of astrocytic Ca^2+^ transients, show depressive-like behaviors, aberrant brain-wide functional connectivity, and reduced frontal-limbic resting-state connectivity, all resembling alterations observed in MDD patients [140]. Further linking Ca^2+^ signaling with inflammation, systemic LPS exposure triggers astrocyte reactivity via Orai1 calcium channels [141]. Interestingly, astrocyte-specific Orai1 knockout mice exhibit resilience to LPS-induced depressive behaviors, along with reduced pro-inflammatory cytokine expression, suggesting that astrocytic Ca^2+^ channels mediate the intersection of neuroinflammation and mood regulation. These findings support the notion that astrocytic Ca^2+^ signaling is not only a marker of MDD but also a potential therapeutic target. This perspective aligns with the tripartite synapse model, which describes the bidirectional exchange of signals between astrocytes and neurons [142], challenging the traditional neurocentric view [143]. This concept has evolved into the multipartite synapse, incorporating microglia [144] and oligodendrocyte precursor cells (OPCs) [145], reflecting the complex, multicellular coordination of synaptic function [146].

### 4.5. Sex-Specific Astrocytic Stress Responses, Molecular Vulnerabilities, and Their Impact on BBB Integrity in MDD

Interestingly, transcriptomic profiling of postmortem brain samples from female MDD patients has shown altered endothelial gene expression, suggesting sex-specific vulnerabilities in BBB function. Supporting this, chronic social defeat stress (CSDS) in female rodents leads to the loss of Cldn5—a tight junction protein—in the PFC and nucleus accumbens, causing BBB leakage and promoting anxiety- and depression-like behaviors [147]. Sex-dependent differences in astrocytic stress responses further illustrate their complexity. Transcriptomic profiling reveals that male mice exposed to chronic stress upregulate vascular interaction genes such as fibroblast growth factor 2 (FGF2), angiotensinogen (AGT), angiopoietin-1 (ANGPT1), and AQP4 in the PFC, while females downregulate the same transcripts, suggesting sex-specific pathways of glial vulnerability that may contribute to the higher prevalence of depression in women [148]. Other molecular mediators, including fatty acid-binding protein 7 (FABP7), exemplify the diverse roles astrocytes can play. In hippocampal astrocytes, FABP7 overexpression protects against stress-induced depressive-like behaviors by attenuating neuroinflammation, promoting dendritic spinogenesis, and enhancing BBB stability [149]. However, in pathological contexts such as amyotrophic lateral sclerosis (ALS), FABP7 upregulation promotes NF-κB-driven pro-inflammatory signaling and astrocytic neurotoxicity, while its silencing reduces motor-neuron toxicity, underscoring its dual and context-dependent nature [150]. FABP7 also protects astrocytes against oxidative stress by promoting lipid droplet formation and regulates dendritic morphology and excitatory synaptic function, linking astrocytic lipid metabolism to neuronal adaptability [151,152]. Astrocytic transcriptional regulators also shape stress outcomes. In nucleus accumbens astrocytes, cAMP response element-binding protein (CREB) has been identified as a key regulator of stress susceptibility, being upregulated in stress-susceptible mice and downregulated in resilient ones. Viral overexpression of CREB in astrocytes promotes susceptibility and depressive-like behaviors, directly linking astrocytic transcriptional reprogramming to behavioral outcomes [153]. Similarly, zinc finger and BTB domain-containing protein 7A (Zbtb7a) induction shifts astrocytic gene expression away from metabolic support toward inflammatory activity, with its upregulation both necessary and sufficient to produce depressive-like phenotypes [154]. Epigenetic mechanisms add further vulnerability: chronic stress induces histone modifications in astrocytic promoters regulating glutamate transporters, connexins, and calcium signaling, creating long-lasting molecular imprints that persist after stress cessation and may contribute to recurrence of depressive episodes [155].

A summary of the changes in gene and astrocytic protein expression is presented in the table below (Table 1).

## 5. Therapeutic Implications and Future Directions

Recent advances in neuropsychiatric research have increasingly positioned astrocytes as critical therapeutic targets in MDD, shifting attention beyond the traditional focus on neuronal and microglial dysfunction. Astrocytes, once regarded primarily as support cells, are now recognized as active regulators of brain homeostasis. In MDD, they exhibit marked alterations, including reduced density and number, aberrant gene expression, and morphological abnormalities [96,157]. Therapeutic strategies aiming to upregulate EAAT2, such as treatment with β-lactam antibiotics like ceftriaxone, have demonstrated neuroprotective effects in vitro and in vivo by restoring glutamate homeostasis [158]. Astrocytes also appear to be active participants in the pharmacological action of antidepressants. Selective serotonin reuptake inhibitors (SSRIs), the first-line treatment for MDD, are known to interact with astrocytic 5-HT2B receptors, suggesting that astrocytes may contribute directly to their therapeutic efficacy [96,157]. Importantly, antidepressant efficacy depends on astrocytic health: fluoxetine requires intact AQP4 signaling to restore astrocytic morphology and glia–vascular coupling, highlighting astrocytic plasticity as an essential component of treatment response [62].

Astrocytic GRs are more sensitive to stress than neuronal GRs; experimental deletion of these receptors in astrocytes induces depression-like behaviors, while their restoration reverses these effects [67]. Chronic glucocorticoid exposure impairs astrocyte metabolism, reduces neurotrophic support, increases reactivity, and contributes to neuroinflammation [159]. These findings position astrocytic GRs as a promising therapeutic target. Selective GR modulators capable of restoring proper receptor function in astrocytes may help normalize stress responses, reduce inflammation, and re-establish transcriptional control over critical astrocytic pathways such as glutamate transport and calcium homeostasis [160].

In parallel, neuromodulatory techniques such as transcranial magnetic stimulation (TMS) have shown potential to influence astrocyte activity. Although traditionally viewed as targeting neuronal circuits, recent evidence suggests that TMS may also modulate astrocytic calcium signaling, enhance neurotrophic support, and reduce pro-inflammatory cytokine release. TMS has been shown to increase astrocytic expression of BDNF and upregulate EAAT2, potentially reversing stress-induced astrocytic dysfunction [161]. These effects support synaptic repair and improved neuronal-glial communication. Furthermore, tailoring TMS protocols to target cortical regions with pronounced astrocyte pathology may enhance treatment outcomes, reinforcing the therapeutic value of personalized neuromodulation strategies.

Anti-inflammatory agents—including minocycline, ibudilast, and certain non-steroidal anti-inflammatory drugs (NSAIDs)—have demonstrated the capacity to reduce astrocyte-mediated inflammation, suppress NF-κB signaling, and increase BDNF expression. In animal models, minocycline has been shown to normalize astrocytic morphology and mitigate depressive-like behaviors, while ibudilast improves mood-related outcomes by modulating glial activity. Other promising compounds targeting inflammasome pathways or glial metabolism, such as PPAR-γ agonists, may also aid in restoring astrocyte homeostasis, particularly in cases of chronic stress [159]. Ketamine also has therapeutic applications as an NMDA receptor antagonist [156].

Building on these therapeutic insights, recent developments in molecular neuroscience have introduced novel astrocyte-focused strategies that aim to address glial dysfunction at its source. Astrocyte-specific delivery systems, including nanoparticles, adeno-associated viral vectors with astrocyte-tropic promoters, and lipid-based carriers, are being designed to transport therapeutic agents across the BBB with cell-type specificity [162,163]. These platforms enable the localized delivery of drugs that upregulate EAAT2, modulate GR signaling, or deliver anti-inflammatory agents directly to astrocytes, enhancing therapeutic efficacy while minimizing systemic side effects.

Additionally, reprogramming dysfunctional astrocytes represents an emerging therapeutic frontier. Through transcription factor modulation or small-molecule intervention, reactive or senescent astrocytes can be induced to revert to a healthy, supportive state. Some studies even suggest the possibility of directly converting astrocytes into functional neurons, providing a potential strategy for repairing damaged circuits in both depression and comorbid neurodegenerative diseases.

Epigenetic regulation, particularly through miRNAs, is also gaining recognition as a crucial modulator of astrocyte function in depression. miRNA-based therapies aim to either inhibit pathological miRNAs or deliver protective ones using viral vectors, synthetic oligonucleotides, or exosome-mediated transfer [164,165]. These interventions offer high specificity in correcting astrocyte gene expression without affecting other cell types and hold considerable promise as next-generation treatments for MDD.

Taken together, this growing body of evidence supports a fundamental shift in the treatment paradigm for depression. Rather than focusing exclusively on neurons, emerging strategies emphasize the central role of astrocytes in regulating brain function and resilience to stress. Targeting astrocytic dysfunction through EAAT2 upregulation, GR modulation, anti-inflammatory agents, neuromodulation, and advanced molecular tools offers not only symptom relief but the potential to modify the underlying disease trajectory. These astrocyte-centered therapies are especially promising for treatment-resistant and inflammation-driven subtypes of MDD, paving the way toward a new era of precision glial medicine in psychiatry.

## 6. Conclusions

Depression remains a major global health challenge, and while its multifactorial nature has long been acknowledged, the critical role of astrocytes in its pathophysiology has only recently gained well-deserved attention. This review underscores how chronic stress, a well-established precipitant of MDD, induces a cascade of molecular and cellular changes that directly impair astrocyte function. Through mechanisms involving dysregulation of GR signaling, neuroinflammation, glutamate toxicity, oxidative stress, and epigenetic modifications, astrocytes shift from supportive regulators of neural homeostasis to dysfunctional contributors to neuropathology.

Astrocytes are not passive bystanders in brain function—they actively shape synaptic transmission, maintain BBB integrity, regulate neurotrophic support, and modulate the emotional and cognitive circuits disrupted in depression. The emerging evidence from transcriptomic and epigenetic studies, particularly in postmortem human tissue and chronic stress animal models, reveals consistent patterns of astrocyte-specific gene expression changes—highlighting both potential biomarkers and therapeutic targets.

Despite compelling progress, several important limitations constrain current interpretations. First, much of the mechanistic evidence derives from rodent models, which, although invaluable, do not fully capture the complexity of human astrocyte biology. Human astrocytes differ in size, heterogeneity, calcium signaling dynamics, and transcriptional profiles; these distinctions raise questions about how faithfully rodent findings translate to the human condition. Second, postmortem human studies, while crucial for understanding MDD-related glial pathology, are limited by small sample sizes, variability in medication status, comorbidities, and the inability to track temporal changes in astrocyte function. These constraints complicate causal inferences about whether observed astrocytic abnormalities represent drivers, compensatory adaptations, or downstream consequences of prolonged stress and depression.

Another limitation lies in the heterogeneity of astrocyte subtypes, which is only beginning to be resolved with single-cell transcriptomics. Many studies still treat astrocytes as a uniform population, overlooking region-specific and state-dependent differences that may explain resilience versus vulnerability to stress. In addition, sex differences in astrocyte responses remain underexplored, despite the markedly higher prevalence of MDD in women and known sex-specific patterns in stress reactivity and neuroinflammation.

Methodologically, the field also lacks longitudinal, in vivo approaches that follow astrocyte dysfunction from the onset of stress exposure through the development of depressive phenotypes. Advances in imaging, human iPSC-derived astrocyte models, and glia-targeted viral tools offer promising avenues to address this gap. Finally, while emerging therapies aim to restore astrocytic support—whether through glutamate regulation, anti-inflammatory interventions, or epigenetic modulation—their specificity and safety in humans remain untested, underscoring the need for rigorous translational research.

In sum, although the evidence positions astrocytes as central mediators of the stress–depression axis, a comprehensive understanding of their contributions requires more nuanced models, human-based studies, and consideration of biological diversity across sex, age, and brain region. Addressing these limitations will be essential for advancing astrocyte-targeted interventions from conceptual promise to clinically effective treatments.

## Figures and Tables

**Table 1:** Changes in the expression of astrocytic genes and proteins in depression.

Gene/Protein	Function	Change in MDD	Evidence/Model	References
SLC1A2/GLT-1	Glutamate transporter (astrocytic glutamate clearance)	↓ Expression (PFC, hippocampus, LC)	Postmortem, neuroimaging, animal models; blockade induces depressive-like behavior	[129]
SLC1A3/GLAST	Astrocytic glutamate transporter	↓ Expression (PFC, hippocampus, LC)	Postmortem and chronic stress models	[156]
GFAP	Structural astrocyte marker	↓ Expression (LC and others)	Postmortem studies, rodent models	[65,120]
GABAA receptor subunits	Inhibitory neurotransmission (β3, δ, γ2, α1, etc.)	↓ Expression (PFC, ACC, hippocampus)	Postmortem (suicidal patients), stress models	[103,105]
MAO-B	GABA synthesis in reactive astrocytes	↑ Activity (FSL rats)	FSL depression model; blockade restores plasticity	[106]
S100Β	Astrocyte calcium-binding protein/injury marker	↑ in CSF/serum, ↓ in brain tissue	Clinical samples	[82]
GRIK2, BEGAIN	Glutamatergic signaling, synaptic plasticity	↓ Methylation → altered expression	Epigenetic analyses in postmortem tissue	[118]
TCF7L2	Astrocyte differentiation and inflammation regulation	↓ Expression (hippocampus)	Mouse models; restored by antidepressants	[119]
Connexins (CX43, CX30)	Gap junction proteins for astrocyte-astrocyte communication	↓ Expression (PFC, hippocampus, LC)	Postmortem and chronic stress animal models	[65,66,123]
Claudin-5 (CLDN5)	Tight junction protein (BBB integrity)	↓ Expression (PFC, Nucleus Accumbens; sex-specific)	CSDS model in female rodents	[147]
Cytokines (IL-1 B, IL-6, TNF-A)	Neuroinflammation	↑ Expression (astrocyte-mediated)	Clinical data and LPS-induced inflammation in models	[82,83]
PMCA2, ITPR2	Astrocytic calcium signaling	PMCA2 ↑ (early stress); Itpr2^−^/^−^ → ↓ Ca^2+^ transients	Mouse models; linked to depressive-like behavior	[111]
ENTPD2	ATP hydrolysis (purinergic signaling)	↓ Expression (gray matter)	Postmortem (suicidal individuals)	[110]
AQP4	Astrocytic water channel, BBB and K^+^ regulation	↓ Expression (PFC, hippocampus, LC)	Postmortem, CMS/CUS models, knockout studies	[122,125]
GAT-1, GAT-3	Astrocytic GABA uptake; regulates inhibitory tone	Impaired uptake; altered inhibitory signaling	Preclinical stress models	[22]
FABP7	Lipid metabolism, oxidative protection, BBB stability	↑ Protective in stress; ↑ pro-inflammatory in ALS context	Stress models; ALS models	[70,71,72,73]
BDNF	Trophic support; plasticity; emotional regulation	↓ Astrocytic trophic signaling in stress and MDD	Hippocampus, PFC studies	[45,46]
Zbtb7a	Switch between metabolic vs. inflammatory astrocyte state	↑ Expression → depressive-like phenotype	Overexpression studies	[75]
Astrocytic glucocorticoid receptor	Integrates stress signals, regulates metabolism	↓ Expression in MDD; impaired ATP release and glucose metabolism	Postmortem, iPSC, conditional knockout	[66,67,68]

Explanation of symbols: ↑—increase, ↓—decrease.

## Data Availability

No new data were created or analyzed in this study.

## Abbreviations

The following abbreviations are used in this manuscript:

MDD	Major Depressive Disorder
HPA	Hypothalamic–Pituitary–Adrenal
MDEs	Major Depressive Episodes
CNS	Central Nervous System
NVU	Neurovascular Unit
BBB	Blood–Brain Barrier
EAAT1	Excitatory Amino Acid Transporter 1
EAAT2	Excitatory Amino Acid Transporter 2
GLT-1	Glutamate Transporter 1
GLAST	Glutamate Aspartate Transporter
SLC1A3	Solute Carrier Family 1 Member 3
SLC1A2	Solute Carrier Family 1 Member 2
GABA	Gamma-Aminobutyric Acid
GAT-1	Gaba Transporter 1
GAT-3	Gaba Transporter 3
ATP	Adenosine Triphosphate
LTP	Long-Term Potentiation
LTD	Long-Term Depression
NMDA	N-Methyl-D-Aspartate
CB1R	Type 1 Cannabinoid Receptors
OT	Oxytocin
OTRs	Oxytocin Receptors
CeA	Central Amygdala
TGOT	Threonine-4, Glycine-7 Oxytocin
ANLS	Astrocyte–Neuron Lactate Shuttle
MCT1	Monocarboxylate Transporter 1
MCT4	Monocarboxylate Transporter 4
cAMP	Cyclic Adenosine 3′,5′-Monophosphate
PKA	Protein Kinase A
PGE_2_	Prostaglandin E_2_
EETs	Epoxyeicosatrienoic Acids
NO	Nitric Oxide
GDNF	Glial-Derived Neurotrophic Factor
AQP4	Aquaporin-4
BDNF	Brain-Derived Neurotrophic Factor
NGF	Nerve Growth Factor
CNTF	Neurotrophic Factor
TSP-1	Thrombospondin 1
TSP-2	Thrombospondin 2
ANG-1	Angiopoietin-1
SHH	Sonic Hedgehog
RA	Retinoid Acid
IGF-1	Insulin-Like Growth Factor 1
APOE	Apolipoprotein E
GSH	glutathione
Nrf2	nuclear factor erythroid 2–related factor 2
SOD1/2	superoxide dismutases
GPx	glutathione peroxidase
GSTs	glutathione-S-transferases
ROS	Reactive Oxygen Species
GRs	Glucocorticoid Receptors
IL-1β	Interleukin-1β
IL-6	Interleukin-6
TNF-α	Tumor Necrosis Factor-A
IL-1α	Interleukin-1α
mPFC	Medial Prefrontal Cortex
CA3	Cornu Ammonis Area 3
GFAP	Glial Fibrillary Acidic Protein
FGF2	Fibroblast Growth Factor 2
AGT	Angiotensinogen
ANGPT1	Angiopoietin-1
FABP7	Fatty Acid-Binding Protein 7
ALS	Amyotrophic Lateral Sclerosis
NF-κB	Nuclear Factor Kappa-Light-Chain-Enhancer of Activated B Cells
CREB	Camp Response Element-Binding Protein
Zbtb7a	Zinc Finger and Btb Domain-Containing Protein 7a
C1q	Complement Component 1q
Kir4.1	Inwardly Rectifying Potassium Channel 4.1
CUMS	Chronic Unpredictable Mild Stress
SGK1	Serum- And Glucocorticoid-Regulated Kinase 1
FOXO3a	Forkhead Box O3a
LKB1	Liver Kinase B1
HDAC5	Histone Deacetylase 5
AMPK	Amp-Activated Protein Kinase
STAT3	Signal Transducer and Activator of Transcription 3
SCI	Spinal Cord Injury
Cldn5	Claudin-5
ZO-1	Zonula Occludens-1
ER	Endoplasmic Reticulum
mPTP	Mitochondrial Permeability Transition Pore
miRNAs	Micrornas
miR-124	Microrna-124
GSK3β	Glycogen Synthase Kinase 3 Beta
LHPA	Limbic–Hypothalamic–Pituitary–Adrenal
PFC	Prefrontal Cortex
LC	Locus Coeruleus
CMS	Chronic Mild Stress
CUS	Chronic Unpredictable Stress
ACC	Anterior Cingulate Cortex
FSL	Flinders Sensitive Line
MAO-B	Monoamine Oxidase-B
GRIK2	Glutamate Ionotropic Receptor Kainate Type Subunit 2
BEGAIN	Brain-Enriched Guanylate Kinase-Associated Protein
LPS	Lipopolysaccharide
OPCs	Oligodendrocyte Precursor Cells
KO	Knockout
CSF	Cerebrospinal Fluid
SSRIs	Selective Serotonin Reuptake Inhibitors
TMS	Transcranial Magnetic Stimulation
NSAIDs	Non-Steroidal Anti-Inflammatory Drugs
